# Predictive value of heart rate deceleration capacity on coronary artery lesion in acute phase of Kawasaki disease

**DOI:** 10.1038/s41598-020-67121-3

**Published:** 2020-06-23

**Authors:** Yaheng Lu, Yonghong Guo, Feifei Si, Tingting Chen, Mei Jin, Yizhou Wen, Xianmin Wang

**Affiliations:** 1 Department of Pediatric Cardiology, Chengdu Women’s and Children’s Central Hospital, School of Medicine, University of Electronic Science and Technology of China, Chengdu, 611731 China; 2 Women’s and children’s Hospital affiliated to Chengdu Medical College. Sichuan Women’s and Children’s Hospital, Chengdu, 610045 China

**Keywords:** Risk factors, Vasculitis

## Abstract

This study was to investigate the correlation of vagal activity with coronary artery lesion (CAL) in Kawasaki disease (KD) children, and assess the predictive value of heart rate deceleration capacity (DC) for CAL in acute phase of KD.50 KD children with CAL, 130 KD children without CAL, 30 children with acute upper respiratory infection and 100 healthy children were recruited and indicators reflecting vagal activity including DC were measstuogram. KD children with CAL showed decreased vagal activity with significantly lower values of DC. DC was negatively correlated with levels of N-terminal pro-brain natriuretic peptide (NT-proBNP) and C-reactive protein (CRP) in KD children. DC was a usable cardiac electrophysiological index to predict CAL in children with KD, with an area under the receiver operating characteristic curve (AUC) of 0.741. The cut-off value of DC for predicting CAL in KD children was 4.37 ms. DC was an independent predictor of CAL in children with KD, evaluated by multiple logistic regression analysis, KD children with DC ≤ 4.37 ms had an increased risk of CAL, with odds ratios (OR) of 5.94. Our study illustrates DC could be used to predict CAL in acute phase of KD.

## Introduction

Kawasaki disease (KD) is an acute febrile systemic vasculitis syndrome, which is one of the most common acquired heart disease in children^1^. Coronary artery lesion (CAL) as one of the most serious complications, which could result in coronary artery aneurysm, even myocardial infarction and sudden death, that cause serious damage to children’s health, occurs in 25% of untreated children with KD^2–4^. Studies have shown that some biochemical indicators are associated with CAL in KD, but there are relatively few reliable indicators for predicting CAL in the acute phase of disease^5–8^.

Heart rate variability (HRV) reflects beat-to-beat changes in RR intervals, which is a traditional, non-invasive method detecting autonomic nervous function. Root mean squared successive difference (rMSSD), percentage of successive normal sinus RR intervals >50 ms (PNN50) belongs to the time–domain variables of HRV, high frequency (HF) belongs to the frequency domain variable of HRV, low values for these indicators indicate hypoactivity of the vagal nerve^9–11^. Decreased vagal activity has been found in patients with coronary artery disease in adults by HRV analysis, which is closely related to the prognosis and the severity of CAL^12–14^. Since HRV method cannot accurately distinguish between the vagal and sympathetic activities of the autonomic nervous system, Heart rate deceleration capacity (DC) as a new indicator, which reflects the general trend of sinus rhythm RR interval and its ability to decelerate, has been discovered that can more accurately and quantitatively evaluate vagal activity and has a better warning ability of adverse cardiac events than HRV^15–18^. In this study, we aimed to investigate the correlation of vagal activity with CAL in KD children, and assess the predictive value of DC for CAL in acute phase of KD.

## Material and Methods

### Study subjects

Between June 2017 and June 2019,180 children with KD treated at Chengdu Women’s and Children’s Central Hospital, School of Medicine, University of Electronic Science and Technology of China(UESTC) were recruited. 50 cases of KD children with CAL–30 boys and 20 girls, aged from 7 to 49 months old–were assigned into KD-CAL group, while 130 KD cases of KD children without CAL–80 boys and 50 girls, aged from 7 to 61 months old–were assigned into KD-NCAL group. The diagnosis of KD was established according to the American Heart Association guideline in 2017^19^. The echocardiography was performed by a pediatric cardiologist to detect CAL within 30 days after onset of kawasaki disease, at the peak period for CAL^20^. CAL was defined as a coronary artery internal dimeter with a z score of ≥2.5 in at least one of the following coronary arteries: right, left anterior descending, and left main^21^. 100 healthy children– 65 boys and 35 girls, aged from 7 to 51 months old– were recruited as control group. 30 cases of children with fever due to acute upper respiratory tract infection (AURI) –16 boys and 14 girls, aged from 10 to 52 months old–were recruited into AURI group.

Excluded from this study were children with the following conditions: presence of non-sinus rhythm (eg, atrial fibrillation, atrial flutter, sick sinus syndrome or atrioventricular block); complicated with other diseases affecting the autonomic nerve function (eg, hyperthyroidism, anemia, or suffocation); using medications that may affect HRV(eg, cardiotonic, antagonists of the renin-angiotensin system, vasodilators, beta-adrenergic antagonists, neuroleptics, antidepressants, antihistamines, anaesthetic, hormones); other organic heart disease.

All procedures performed in studies involving human participants were in accordance with the ethical standards of the Ethics Committee of Chengdu Women’s and Children’s Central Hospital, School of Medicine, UESTC and with the 1964 Helsinki declaration and its later amendments or comparable ethical standards. Informed consent was obtained from guardians of all individual participants included in the study.

### Dynamic electrocardiogram examination

Children with KD received a 24-hour dynamic electrocardiogram examination after the diagnosis and before intravenous immunoglobulins (IVIG) therapy. Other children were examined within 24 hours after enrollment. The recorded data were analyzed using an offline DMS dynamic electrocardiogram analysis system (DM Software, Stateline, NV, USA). DC and indicators of heart rate variability associated with vagal activity PNN50, rMSSD and HF were calculated automatically.

### Plasma NT-proBNP, CRP detection

Fasting peripheral venous blood 3 ml was drawn in all children except the healthy controls on the next morning after enrollment. The blood samples were collected in EDTA anti-coagulant tubes, then the plasma was segregated for detection. The N-terminal pro-brain natriuretic peptide (NT-proBNP) was detected using Nano-Checker 710 immunochromatographic detector (Nano-Ditech Corporation, USA). The normal value of NT-proBNP is 0~500 pg/ml. The C-reactive protein (CRP) was detected using QuikRead CRP quantitative analyzer (Orion Diagnostica company, Finland). The normal value of CRP is 0~1 mg/dL.

### Statistical analysis

Data was analyzed using SPSS version 19.0 software (IBM Corporation, Armonk, NY, USA). We used the Shapiro-Wilks test to check the distribution of variables and the Levene test to check homogeneity of variances. Because of the non-normal distribution and heterogeneity of variance of some measures, data were presented as the median with the interquartile range for continuous variables. Comparisons among groups were evaluated using the Kruskal-Wallis test, while the two groups were compared by the Mann-Whitney U test. χ^2^ tests were performed to compare categorical variables. All tests were performed as two-sided, the value of *P* < 0.05 was considered to indicate statistical significance. Spearman’s partial correlation was used to assess correlations between variables. To evaluate the performance of different indicators in discriminating CAL in KD, KD children with and without CAL were pooled, the area under the receiver operating characteristic curve (AUC) analysis was performed. The sensitivity and specificity were calculated, and the cutoff value was determined by the Youden index. Multivariate logistic regression was used to identify the independent predictor of CAL in KD children.

## Results

### Comparison of baseline characteristics among groups

Table 1 shows the differences of baseline characteristics among different groups. There were no significant differences in age (*P* = 0.250) and gender ratio (*P* = 0.703) among the groups. The values of DC, PNN50, RMSSD and HF were significantly lower in KD-NCAL group compared with the control group (all *P* < 0.001). The values of DC (*P* = 0.021), PNN50 (*P* = 0.016), and HF (*P* < 0.001) were also lower in AURI group than the control group, while rMSSD was not different. There were no significant differences on the values of DC (*P* = 0.683), PNN50 (*P* = 0.268), RMSSD (*P* = 0.169) and HF (*P* = 0.188) between AURI group and KD-NCAL group. However, The values of DC (*P* < 0.001), PNN50 (*P* = 0.001), RMSSD (*P* = 0.027) and HF (*P* = 0.008) were lower in KD-CAL group compared with AURI group, and were also significantly lower than the control group and (all *P* < 0.001). The values of DC (*P* < 0.001), PNN50 (*P* = 0.001) in KD-CAL group were lower than KD-NCAL group, but RMSSD (*P* = 0.120), HF (*P* = 0.056) were not different between the two groups. The levels of plasma NT-proBNP and CRP were significantly different across different groups with the following rank order: KD-CAL group> KD-NCAL group > AURI group (all *P* < 0.001). Spearman’s partial correlation showed that both DC and PNN50 negatively correlated with NT-proBNP (*r* = −0.883, −0.430, *P* < 0.001), and CRP (*r* = −0.846, −0.377, *P* < 0.001).

**Table 1:** Baseline characteristics of the participants.

Parameter	control group	AURI group	KD-NCAL group	KD-CAL group	F/χ2	P
	(n = 100)	(n = 30)	(n = 130)	(n = 50)		
Age (months)	31 (20~40)	30 (17~45)	33 (20~43)	28 (18~38)	4.101	0.250
Gender male	65	16	80	30	1.410	0.703
female	35	14	50	20		
DC(ms)	6.06 (5.23~6.69)	5.43 (4.76~6.23)^a^	5.47 (4.77~5.95)^a^	3.70 (2.68~5.37)^a, b, c^	60.027	<0.001
PNN50(%)	16 (8~28)	10 (5~19)^a^	9 (2~16)^a^	3 (0~10)^a, b, c^	56.470	<0.001
rMSSD(ms)	45 (33~58)	42 (30~55)	37 (29~45)^a^	32 (26~42)^a, b^	21.507	<0.001
HF(ms^2^)	393.7(226.1~572.6)	146.4 (82.5~229.9)^a^	118.5 (54.1~202.0)^a^	70.3 (40.7~190.7)^a, b^	123.624	<0.001
NT-proBNP (pg/ml)	—	105 (74~120)	398 (118~819)^b^	867 (709~987)^b, c^	65.583	<0.001
CRP (mg/dl)	—	1.5 (0.9~1.9)	3.9 (2.8~5.1)^b^	6.3 (3.9~12.7)^b, c^	88.325	<0.001

Data are presented as medians (interquartile ranges).

^a^*P* < 0.05, compared with the control group; ^b^*P* < 0.05, compared with the AURI group; ^c^*P* < 0.05, compared with the KD-NCAL group; —, this data was not detected; AURI, acute upper respiratory infection; KD, kawasaki disease; CAL, coronary artery lesion; KD-CAL, KD with CAL group; KD-NCAL group, KD without CAL group; DC, heart rate deceleration capacity; PNN50, percentage of successive normal sinus RR intervals >50 ms; HF, high frequency; rMSSD, root mean squared successive difference; NT-proBNP, N-terminal pro-brain natriuretic peptide; CRP, C-reactive protein.

### ROC analysis of using different indicators to predict CAL in children with KD

**Figure 1:**
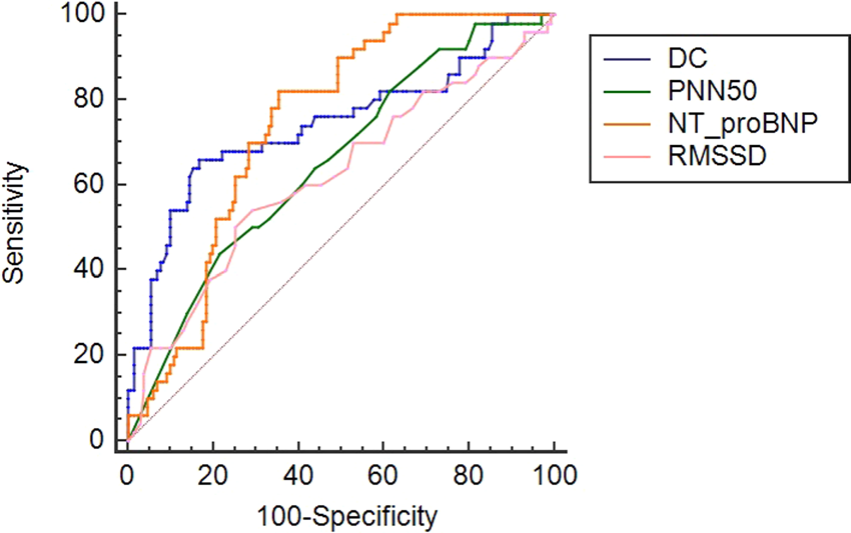
Receiver operating characteristic curves using DC, PNN50, NT-proBNP and CRP to predict CAL in children with KD. CAL, coronary artery lesion; KD, kawasaki disease DC, heart rate deceleration capacity; PNN50, percentage of successive normal sinus RR intervals >50 ms; NT-proBNP, N-terminal pro-brain natriuretic peptide; CRP, C-reactive protein.

The ROC curves of using DC, PNN50, NT-proBNP and CRP to predict CAL in children with KD were analyzed (Fig. 1; Table 2). AUCs corresponding to DC and NT-proBNP, which may be used to predict CAL in children with KD, were demonstrated to be relatively higher (0.741 and 0.744, respectively) compared with PNN50 and CRP. By contrast, the sensitivities of NT-proBNP were revealed to be higher, with values of 82.00% at its cutoff points at 684 pg/ml, compared with that of 66.00, 44.00 and 66.00% for DC, PNN50, and CRP at their classical cutoff points at 4.37 ms, 1%, 5.3 mg/dl, respectively. In comparison, the specificity of DC was determined to be high, with a value of 83.08% at its classical cutoff point, compared with that of 78.46%, 64.62%, 77.69% for PNN50, NT-proBNP, and CRP respectively.

**Table 2:** A summary of receiver operating characteristic curve analysis using DC, PNN50, NT-proBNP and CRP to predict CAL in children with KD.

Indicator	AUC	P-value	95% CI	Cut-off	Sensitivity (%)	Specificity (%)	Youden index
DC	0.741	<0.001	0.671~0.803	4.37^a^	66.00	83.08	0.4908
PNN50	0.654	0.001	0.579~0.723	1 ^b^	44.00	78.46	0.2246
NT-proBNP	0.744	<0.001	0.674~0.806	684 ^c^	82.00	64.62	0.4662
CRP	0.597	0.047	0.522~0.670	53 ^d^	66.00	77.69	0.4369

^a^Values are ms. ^b^Values are %. ^c^Values are pg/ml. ^d^Values are mg/dl. AUC, area under curve; DC, heart rate deceleration capacity; PNN50, percentage of successive normal sinus RR intervals >50 ms; NT-proBNP, N-terminal pro-brain natriuretic peptide; CRP, C-reactive protein.

### Logistic regression analysis of Kawasaki disease with CAL

Using DC ≤ 4.37 ms, PNN50 ≤ 1%, NT-proBNP > 684 pg/ml and CRP > 5.3 mg/dl as binary independent variable, multivariate Logistic regression for CAL in acute phase of Kawasaki disease was analyzed is presented in Table 3.The results showed that DC ≤ 4.37 ms (*P* = 0.024) and NT-proBNP > 684 pg/ml (*P* = 0.032) are independent predictors of CAL in KD children.

**Table 3:** A multivariate Logistic regression model for coronary artery lesion in acute phase of Kawasaki disease.

Indicator	P	OR	95% CI
DC ≤ 4.37 ms	0.024	3.275	1.170~9.173
PNN50 ≤ 1%	0.690	1.195	0.498~2.866
NT-proBNP > 684 pg/ml	0.032	2.968	1.097~8.028
CRP > 5.3 mg/dl	0.195	1.876	0.725~4.851

CI, confidence interval, OR, odds ratio, DC, heart rate deceleration capacity; PNN50, percentage of successive normal sinus RR intervals >50 ms; NT-proBNP, N-terminal pro-brain natriuretic peptide; CRP, C-reactive protein.

## Discussion

Previous studies have found that decreased vagal activity assessed by HRV analysis is associated with myocardial infarction developed from giant coronary artery aneurysm in KD^22^. However, there are few studies on the relationship between vagal activity and CAL in KD. In our study, the recruited KD children were in the acute phase and had fever. Studies have shown that fever can cause a decrease in vagal activity^23,24^, so in addition to the healthy control, children with fever due to AURI were set as the febrile control. In this study, we observed that DC, PNN50 and HF in AURI children and KD children with or without CAL were significantly reduced compared with the healthy children, indicating that vagal activity was decreased in febrile children. Besides, we found DC, PNN50, RMSSD and HF were not significantly different between AURI children and KD children without CAL, indicating that there was no difference in vagal activity between children with different febrile illness. Vagal activity measurements may not be applicable to the discrimination of febrile diseases such as AURI and KD. Nevertheless, we still found that DC, PNN50, rMSSD and HF were lower in KD children with CAL compared with AURI children. DC and PNN50 in KD children with CAL were also lower than those without CAL, though no statistical differences were found in RMSSD and HF between the two groups. It suggests that decreased vagal activity may be associated with CAL in KD children, DC and PNN50 may be more reliable in KD children for the assessment of vagal activity compared with RMSSD, HF.

Exaggerated inflammation in the acute phase that leads to dysfunction of vascular endothelial cells is an important mechanism for the occurrence of CAL^25^. NT-proBNP is an important cardiac biomarker that associated with ventricular myocyte ischemia and hypoxia^26^. CRP is a pentameric protein that is present in the bloodstream during inflammatory events, can reflect the severity of inflammation^27^. Studies have confirmed that levels of NT-proBNP and CRP exist a positive correlation with CAL in KD^5,6^. Recent studies have found that increased vagal activity can reduce the levels of inflammatory factors by activating the “cholinergic anti-inflammatory pathway”, so as to improve endothelial dysfunction^28,29^. In animal and human studies, levels of NT-proBNP and CRP were decreased in response to vagus nerve stimulation^30–33^. In our study, compared to KD-CAL group, relatively higher values of DC and PNN50, lower levels of NT-proBNP and CRP were found in KD-NCAL group. This indicated that KD children without CAL had higher vagal activity and a relatively lower level of inflammation compared with KD children with CAL. At the same time, correlation study showed that DC and PNN50 were negatively correlated with levels of plasma NT-proBNP and CRP in KD children. It is speculated that increasing vagal activity in KD children could be a way of protection of the coronary artery by reducing the production of inflammatory factors.

In the present study, DC cutoff value of 4.37 ms yielded sensitivity of 66.00% and specificity of 83.08% for predicting CAL, KD children with DC ≤ 4.37 ms (OR = 3.275) had an increased risk of CAL. In adults, it has been demonstrated that DC < 2.5 ms is significantly related to a higher risk of death after myocardial infarction, while DC > 4.5 ms is related to a lower risk^16,34^. Wei Hu’s research found that DC was an independent risk factor for heart failure, with cutoff values of 4.55 ms in males and 4.85 ms in females^35^. Although the cutoff values of DC are varied from study to study in different prediction events, these studies suggest that DC is a useful prognostic tool for risk stratification in cardiovascular diseases. NT-proBNP is also a strong predictor of CAL in KD children. Kaneko *et al*. reported that the NT-proBNP cutoff value of 1000 pg/ml yielded sensitivity of 68% and specificity of 83% for predicting CAL^8^. Yoshimura *et al*. indicated that a cutoff value of 1300 pg/ml to predict CAL produced specificity of 95% and sensitivity of 85%^36^. In our study, the cutoff value was 684 pg/ml, which yielded sensitivity of 82.00% and specificity of 64.62%. The relatively lower value of NT-proBNP in our study may have occurred because we performed the test during the acute phase of KD. KD children with NT-proBNP >684 pg/ml (OR = 2.968) had a higher risk of CAL. The odds ratio of NT-proBNP is lower compared with that of DC. It is suggested that the relevance of DC and CAL is stronger compared with NT-proBNP.

This study has some limitations. First, the sample size of this study is small. Large-scale, multicenter prospective studies are needed to confirm our findings. Second, we excluded patients with non-sinus rhythm in this study. Therefore, our findings may not be directly generalized to these patients. Third, temperature fluctuations have an effect on vagal activity. HRV and DC after body core temperature correction were more reliable in studying the correlation between vagal activity and CAL in KD. Unfortunately, we did not have the equipment for continuous monitoring of body temperature and dynamic electrocardiogram at the same time.

In conclusion, decreased vagal activity may be associated with CAL in KD children, the DC would provide an insight into the disease severity and could be used as an independent predictor for the risk of CAL in acute phase of KD.

## References

[1] Wood LE, Tulloh RMR (2009). Kawasaki disease in children. Heart.

[2] Kuo HC (2017). Preventing coronary artery lesions in kawasaki disease. Biomed J..

[3] Duan C, Du ZD, Wang Y, Jia LQ (2014). Effect of pravastatin on endothelial dysfunction in children with medium to giant coronary aneurysms due to kawasaki disease. World J Pediatr.

[4] Holve TJ (2014). Long-term cardiovascular outcomes in survivors of kawasaki disease. Pediatrics.

[5] Min KK, Min SS, Gi BKF (2018). Factors predicting resistance to intravenous immunoglobulin treatment and coronary artery lesion in patients with kawasaki disease: analysis of the korean nationwide multicenter survey from 2012 to 2014. Korean Circ J.

[6] Jun H (2016). Age-adjusted plasma N-terminal pro-brain natriuretic peptide level in kawasaki disease. Korean J Pediatr.

[7] Kobayashi T (2006). Prediction of intravenous immunoglobulin unresponsiveness in patients with kawasaki disease. Circulation.

[8] Kaneko K (2011). Prediction of the risk of coronary arterial lesions in kawasaki disease by brain natriuretic peptide. Pediatr Cardiol.

[9] Ewing DJ, Borsey DQ, Bellavere F, Clarke BF (1981). Cardiac autonomic neuropathy in diabetes: comparison of measures of R-R interval variation. Diabetologia.

[10] Paiva VC (2011). Comparison of assessment methods of cardiac vagal modulation. Arq Bras Cardiol.

[11] Bigger JT, Fleiss JL, Rolnitzky LM, Steinman RC (1993). Frequency domain measures of heart period variability to assess risk late after myocardial infarction. J Am Coll Cardiol.

[12] Yıldız BS (2016). Evaluation of heart rate variability in patients with coronary artery ectasia and coronary artery disease. Turk Kardiyol Dern Ars.

[13] Wennerblom B, Lurje L, Tygesen H, Vahisalo R, Hjalmarson A (2000). Patients with uncomplicated coronary artery disease have reduced heart rate variability mainly affecting vagal tone. Heart.

[14] Li HR (2016). Additive value of heart rate variability in predicting obstructive coronary artery disease beyond framingham risk. Circ J.

[15] Hamm W (2018). Deceleration capacity of heart rate after acute altitude exposure. High Alt Med Biol.

[16] Bauer A (2006). Deceleration capacity of heart rate as a predictor of mortality after myocardial infarction: cohort study. Lancet.

[17] Pan Q (2016). The degree of heart rate asymmetry is crucial for the validity of the deceleration and acceleration capacity indices of heart rate: a model-based study. Comput Biol Med.

[18] Guzik P (2012). Heart rate deceleration runs for postinfarction risk prediction. J Electrocardiol.

[19] McCrindle BW (2017). Diagnosis, treatment, and long-term management of kawasaki disease: a scientific statement for health professionals from the american heart association. Circulation.

[20] Burns JC, Matsubara T (2018). New insights into cardiovascular disease in patients with kawasaki disease. Curr Opin Pediatr.

[21] McCrindle BW (2007). Coronary artery involvement in children with kawasaki disease: risk factors from analysis of serial normalized measurements. Circulation.

[22] Kikuchi Y, Sato Y, Ichihashi K, Shiraishi H, Momoi MY (2003). Autonomic function in kawasaki disease with myocardial infarction: usefulness of monitoring heart rate variability. Pediatr Int.

[23] Kinugasa H, Hirayanagi K (1999). Effects of skin surface cooling and heating on autonomic nervous activity and baroreflex sensitivity in humans. Exp Physiol.

[24] Massaro AN (2017). Effect of temperature on heart rate variability in neonatal ICU patients with hypoxic-ischemic encephalopathy. Pediatr Crit Care Med.

[25] Agarwal S, Agrawal DK (2017). Kawasaki disease: etiopathogenesis and novel treatment strategies. Expert Rev Clin Immunol.

[26] Heck PB, Müller J, Weber R, Hager A (2013). Value of N-terminal pro-brain natriuretic peptide levels in different types of fontan circulation. Eur J Heart Fail.

[27] Hwang J, Seo Y, Jo Y, Son J, Choi J (2016). Aptamer-conjugated live human immune cell based biosensors for the accurate detection of c-reactive protein. Sci Rep.

[28] Zhao M (2013). Vagal stimulation triggers peripheral vascular protection through the cholinergic anti-inflammatory pathway in a rat model of myocardial ischemia/reperfusion. Basic Res Cardiol.

[29] Moser M (2017). Investigation of a micro-test for circulatory autonomic nervous aystem responses. Front Physiol.

[30] Lorgis L (2012). High N-terminal pro-B-type natriuretic peptide levels are associated with reduced heart rate variability in acute myocardial infarction. PLoS ONE.

[31] Rauchenzauner M (2008). N-terminal pro-brain natriuretic peptide (NT-proBNP) release in children with vagus nerve stimulation. A prospective case series. J Neurol.

[32] Yu H (2014). Chronic vagus nerve stimulation improves left ventricular function in a canine model of chronic mitral regurgitation. J Transl Med.

[33] Sloan RP (2007). RR interval variability is inversely related to inflammatory markers: the CARDIA study. Mol Med Camb Mass.

[34] Rizas KD (2018). Bedside autonomic risk stratification after myocardial infarction by means of short-term deceleration capacity of heart rate. Europace.

[35] Hu W (2016). Deceleration and acceleration capacities of heart rate associated with heart failure with high discriminating performance. Sci Rep.

[36] Yoshimura K (2013). N-terminal pro-brain natriuretic peptide and risk of coronary artery lesions and resistance to intravenous immunoglobulin in kawasaki disease. J Pediatr.

